# The Involvement of Notch1-RBP-Jk/Msx2 Signaling Pathway in Aortic Calcification of Diabetic Nephropathy Rats

**DOI:** 10.1155/2017/8968523

**Published:** 2017-12-31

**Authors:** Caipan Gong, Li Li, Chunmei Qin, Weihua Wu, Qi Liu, Ying Li, Linwang Gan, Santao Ou

**Affiliations:** ^1^Department of Nephrology, The Affiliated Hospital of Southwest Medical University, Luzhou, Sichuan 646000, China; ^2^Department of Nephrology, Luzhou People's Hospital, Luzhou, Sichuan 646000, China

## Abstract

**Background:**

This study explored the changes in expression of vascular smooth muscle cell (VSMC) markers and osteogenic markers, as well as the involvement of Notch1-RBP-Jk/Msx2 pathway in a rat model of diabetic nephropathy (DN) with vascular calcification.

**Methods:**

A rat model of DN with concomitant vascular calcification was created by intraperitoneal injection of streptozotocin followed by administration of vitamin D3 and nicotine. Biochemical analysis and histological examination of aortic tissue were performed. VSMC markers and osteogenic markers as well as target molecules in Notch1-RBP-Jk/Msx2 were determined by quantitative real-time polymerase chain reaction and immunohistochemical analysis.

**Results:**

Serum calcium and phosphorus levels were significantly increased in model rats as compared to that in normal controls. Diabetic rats with vascular calcification exhibited mineral deposits in aortic intima-media accompanied by decreased expression of VSMC markers and increased expression of osteogenic markers. Notch1, RBP-Jk, Msx2, Jagged1, and N1-ICD were barely expressed in the aortic wall of normal rats. In contrast, these were significantly increased in the model group at all time points (8, 12, and 16 weeks), as compared to that in the normal rats.

**Conclusion:**

Activation of the Notch1-RBP-Jk/Msx2 signaling pathway may be involved in the development and progression of vascular calcification in DN.

## 1. Introduction

Vascular calcification is a key pathological process that contributes to cardiovascular complications of chronic kidney disease (CKD) and is also an independent risk factor for cardiovascular events and mortality in patients with CKD [1, 2]. Diabetic nephropathy (DN) is a leading cause of CKD and is associated with high incidence and quick progression of vascular calcification [3]. About 78% of diabetic patients with preserved kidney function were shown to exhibit varying degrees of vascular calcification in femoral, posterior tibial, and dorsalis pedis arteries [4].

Vascular calcification is a complex, irreversible biological process, which involves differentiation of vascular smooth muscle cells (VSMCs) into chondrocyte- or osteoblast-like cells (chondrogenesis or osteogenesis). It is accompanied by downregulation of contractile VSMC markers, such as alpha-smooth muscle actin (*α*-SMA) and smooth muscle 22 alpha (SM22*α*), and upregulation of various osteogenic transcription factors, such as runt-related transcription factor 2 (Runx2), Msx2, alkaline phosphatase (ALP), osteopontin (OPN), and bone morphogenetic protein 2 (BMP2) [5–8]. Several signaling pathways such as Wnt/*β*-catenin and Notch1-RBP-Jk are believed to be involved in the process of vascular calcification [9, 10]. However, the precise regulatory mechanism of vascular calcification process is not yet clear.

Recent studies have shown that Notch1 signaling pathway, a regulator of angiogenesis and osteogenesis, plays a role in the development of vascular calcification [6, 10, 11]. In mammals, the highly conserved Notch pathway involves four type I transmembrane receptors (Notch1–4), five classical ligands [Jagged 1, 2, and Delta-like (DLL) 1, 3, and 4], and nonclassical ligand DNER, in the regulation of a variety of biological processes, such as cell proliferation, differentiation, and apoptosis [12]. Notch pathway has been extensively studied in the field of tumor growth and targeted therapy [13]; however, its possible involvement in the pathogenesis of diabetic vascular calcification, as well as the specific underlying mechanisms, has rarely been reported.

In this study, we used a modified animal model of vascular calcification in the context of DN by administering a high-fat diet and injection of streptozotocin (STZ), followed by intragastric administration of nicotine and intramuscular injection of vitamin D3. The study explored the changes in expressions of VSMC markers, *α*-SMA and SM22*α*, and osteogenic markers, Runx2 and ALP, and further investigated the relationship between the target molecules in Notch1-RBP-Jk/Msx2 pathway and vascular calcification in aortic tissues in the rat model.

## 2. Methods and Materials

### 2.1. Animal Model

Specific pathogen-free Sprague-Dawley (SD) rats (*n* = 42, male, 4–6 weeks old; weight: 170–220 g) were obtained from the animal center at the Southwest Medical University. The experimental protocol was approved by the ethics committee of the Animal Care and Use Committee at the Southwest Medical University [Permit number, SYSK (CHUAN) 2013-065]. The rats were kept under observation for one week prior to the start of the experiment. They were then randomly divided into two groups, that is, normal controls (Nor group, *n* = 18) and DN rats with vitamin D3/nicotine-induced vascular calcification (DN + VDN group, *n* = 24).

**Figure 1:**
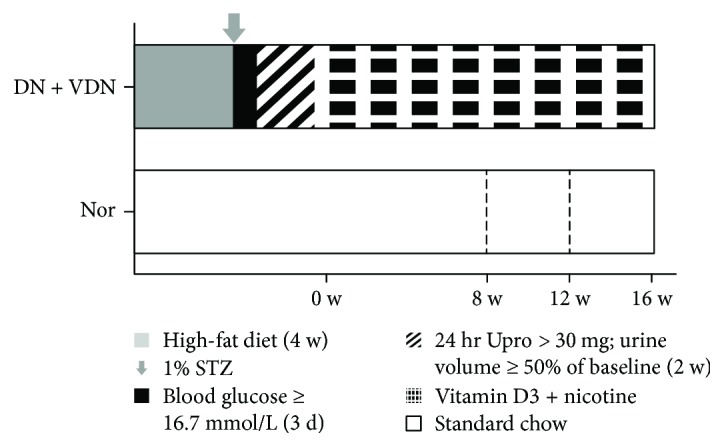
Schematic illustration of the experimental protocol. W: week; STZ: streptozotocin; Upro: urine protein. Nor group: normal controls; DN + VDN group: diabetic nephropathy rats with vitamin D3/nicotine-induced vascular calcification.

Twenty-four SD rats were fed high-fat diet for four weeks. The high-fat diet contained 55% carbohydrate, 10% lard, 10% soybean oil, 11% protein, 2.5% cholesterol, and 11.5% fiber. Following 12 h fasting, the rats were administered a single intraperitoneal injection of streptozotocin, 35 mg/kg (STZ, Sigma Chemical Co., St. Louis, MO, USA) in citrate buffer (1%, *w*/*v*). Three days after the injection of STZ, whole-blood specimens were obtained from mouse tail-vein. Twenty-two rats with blood glucose level > 16.7 mmol/L for 3 consecutive days were deemed to have developed diabetes mellitus. DN was identified based on 24 h urinary protein excretion >30 mg and increase in urine volume by ≥50% from the baseline. Two weeks later, one rat that did not meet the criteria was excluded. After establishment of the DN model, the DN rats were administered intramuscular injection of vitamin D3 (300,000 U/kg) once daily at 9:00 Hrs and intragastric nicotine (Merck, Darmstadt, Germany) dissolved in peanut oil (25 mg/kg, 5 mL/kg) twice daily (at 9:00 and 18:00 Hrs) [14]. The Nor rats were fed standard chow for four weeks, followed by administration of an equal volume of citrate buffer (vehicle). The control rats were administered intramuscular injection of saline and an equal volume of intragastric peanut oil following the same schedule. The rats were sacrificed at 8, 12, and 16 weeks (*n* = 6 at each time point). During the experiment, food and water intake by the rats, their mental state, and blood glucose levels in tail-vein blood were monitored to avoid ketoacidosis or accidental death. The rats were administered subcutaneous insulin injection if the blood glucose level exceeded 26 mmol/L. The timeline of the experimental interventions in the study is shown in Figure 1.

The general conditions of all rats were monitored daily, including mental state, activities, and fur. Body weight was recorded every week throughout the experiment.

24 h urinary protein excretion of diabetic rats was determined at 2 weeks after diagnosis of diabetes and before sacrifice. Successful modeling of DN was confirmed if 24 h urinary protein excretion was more than 30 mg. The rats were fasted for 24 h and then placed in metabolic cages for 24-hour urine collection. Urine protein concentrations were determined by Beckman automatic biochemistry analyzer (Beckman Coulter, Fullerton, CA, USA).

### 2.2. Biochemical and Histological Analysis

After weighing, the rats were anesthetized by an intraperitoneal injection of 2% pentobarbital sodium (Sigma Chemical Co., St. Louis, MO, USA) at a dose of 50 mg/kg and then fixed on an operation table. The abdominal aorta was separated after exposure of the abdominal cavity. The blood was collected from the abdominal aorta and centrifuged at 5000 rpm/min for 10 min at 4°C. The supernatant was collected and labeled and then stored at −20°C until further processing. Serum creatinine (Scr), blood urea nitrogen (BUN), serum calcium (Ca), and phosphorus (P) were measured using an automatic biochemical analyzer.

After blood sample collection, the aorta was resected. The aortic lumen was rinsed with cold saline. The thoracic and abdominal aortic tissues were immersed in 4% formalin solution for at least 24 hours and treated with graded series of ethanol for dehydration and paraffin-embedded, and 4 *μ*m thick sections were prepared. Sections were then stained with von Kossa (Shanghai GenMed, China) and counterstained with Nuclear fast red (Shanghai GenMed, China) to visualize the nuclei. The sections were mounted on glass slides, and three randomly selected fields per slice were examined under an inverted phase contrast microscope (Nikon, Japan).

The degree of vascular calcification was semiquantitatively assessed by two independent observers according to the following scale [15]: 0, no mineral content; 1, a few small dispersed concretions; 2, numerous small dispersed concretions; 3, large granular concretions; and 4, large areas occupied by fused mineral deposits. In case of disagreement, the average of the two scores was considered in the final analysis.

### 2.3. Molecular and Immunohistochemical Analysis

Total RNA was extracted from frozen thoracic aorta tissues of rats using an Eastep® Super kit (Shanghai ProMega, China), and total RNA sample was reverse-transcribed into complementary DNA (cDNA) using the Eastep RT Master Mix (5x) Kit (Shanghai ProMega, China). The sequences of the primers used are shown in Supplementary Table 1. DNA amplification was performed on a StepOne real-time polymerase chain reaction (PCR) system (Eppendorf, Germany) with Eastep qPCR Master Mix (2x) Kit (Shanghai ProMega, China) using the following thermal conditions: an initial step at 95°C for 2 minutes, followed by 40 cycles of 95°C for 15 seconds, 60°C for 1 minute, and 95°C for 15 seconds, and a final phase at 60°C for 5 minutes. The relative amount of mRNA of each sample was calculated using the 2^−ΔΔCT^ method and corrected by reference to the expression of GAPDH (loading control).

The following primary antibodies were used for immunohistochemical analysis: monoclonal rabbit anti-rat RBP-Jk, Msx2, and N1-ICD (Shanghai Bioworld Tech Inc., China); monoclonal rabbit anti-rat Jagged1 and Runx2 (Beijing BioTech Inc., China); monoclonal rabbit anti-rat Notch1 (Wuhan Proteintech Group Inc., China); and monoclonal mouse anti-rat *α*-SMA (Wuhan Boster Bio-Tech., Ltd, China). Immunohistochemical staining was performed according to the manufacturer's instructions. Briefly, paraffin-embedded thoracic aortic tissues were cut into 4 *μ*m thick sections. After antigen retrieval by heating in a microwave, the sections were incubated overnight with primary antibodies at 4°C. After rinsing with phosphate-buffered saline (PBS), the sections were incubated with anti-mouse or anti-rabbit secondary antibodies coupled to horseradish peroxidase (Zhongshan Golden Bridge Biotechnology, Beijing, China). The reaction was visualized using 3, 3′-Diaminobenzidine (DAB, Zhongshan Golden Bridge Biotechnology, China). The sections were rinsed with water and counterstained with Mayer's hematoxylin (Beijing Solarbio Biotech, China). The known positive control in the kit was used as the positive control and replacement of the primary antibody with PBS was used for the negative control. Positive immunostaining was presented as brown or yellow granules in the cytoplasm and/or nucleus. Five visual fields were randomly selected and assessed for immunoreactive areas at 200x magnification using an inverted phase-contrast microscope (Nikon, Japan). The image was analyzed by Image-Pro Plus 6.0 software (Media Cybernetics Inc., Rockville, MD, USA), with color histogram in HSI model (H: 0–30, S: 0–255, I: 0–230); optical density range was 0–6000. The cumulative optical density (IOD) and area of the selected regions were acquired by the software, and the IOD/area was calculated as mean ± standard deviation (SD) for analysis.

### 2.4. Statistical Analysis

All statistical analysis was performed using SPSS17.0 software (SPSS Inc., Chicago, IL, USA). Quantitative data are presented as mean ± SD. The normality of distribution of the continuous quantitative variables was assessed by the Kolmogorov-Smirnov test. Intergroup and intragroup differences were assessed using the independent sample *t*-test and one-way analysis of variance, respectively. Scores of von Kossa staining were analyzed by the Wilcoxon rank-sum test. *p* < 0.05 was considered statistically significant.

## 3. Results

### 3.1. The General Condition of the Rats

Compared with rats in the Nor group, the DN rats exhibited polydipsia, polyphagia, polyuria, loss of body weight, dull fur, and reduced activities at 8 weeks after establishment of the model. Rats in the DN + VDN group showed arched back and piloerection, abdominal bulge, and tail ulcers at 12 weeks, and severe depression at 16 weeks.

**Figure 2:**
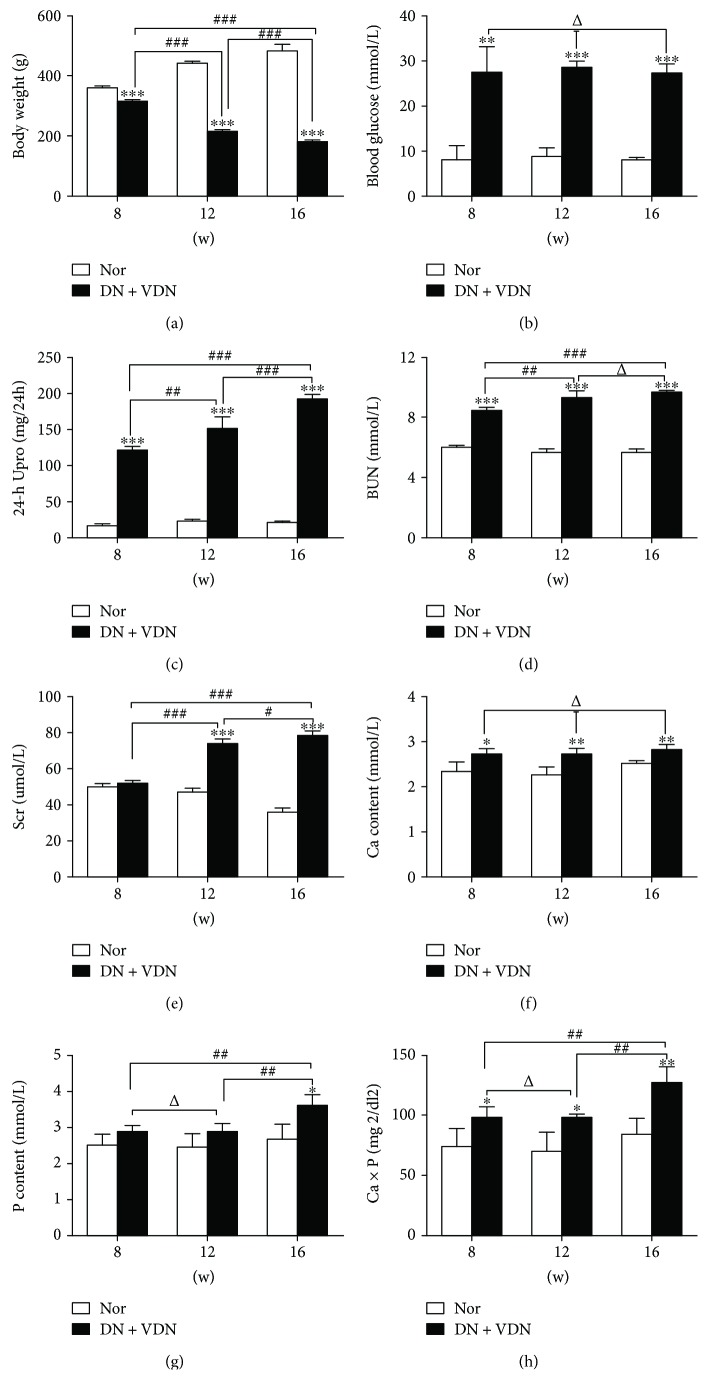
Body weight and various biochemical indicators of rats at 8, 12, and 16 weeks. (a) body weight; (b) blood glucose; (c) 24 h urine protein excretion (24 h Upro); (d) blood urea nitrogen (BUN); (e) serum creatinine (Scr); (f) serum calcium (Ca); (g) serum phosphorus (P); and (h) calcium-phosphorus product (Ca x P). Data presented as mean ± standard deviation (SD). ^∗^*p* < 0.05, ^∗∗^*p* < 0.01, and ^∗∗∗^*p* < 0.001 versus Nor group; ^Δ^*p* > 0.05, ^#^*p* < 0.05, ^##^*p* < 0.01, and ^###^*p* < 0.001. Nor group: normal controls; DN + VDN group: diabetic nephropathy rats with vitamin D3/nicotine-induced vascular calcification.

The body weight of normal rats gradually increased over time. On the contrary, rats in the DN + VDN group lost substantial weight with the progression of diabetes; between-group differences at 8, 12, and 16 weeks were statistically significant (*p* < 0.001, Figure 2(a), Supplementary Table 2). Rats in the DN + VDN group presented remarkable increase in blood glucose level, 24 h urine protein excretion, Scr, and serum BUN levels, as compared to that in the Nor group at 8, 12, and 16 weeks (*p* < 0.01 or *p* < 0.001; Figures 2(b)–2(e); Supplementary Table 2).

In addition, rats in the DN + VDN group exhibited elevated serum phosphorus levels at 16 weeks (*p* < 0.05), elevated serum calcium levels at 8, 12, and 16 weeks (*p* < 0.05 or *p* < 0.01), and resultant increase in calcium-phosphorus product at 8, 12, and 16 weeks (*p* < 0.05 or *p* < 0.01; Figures 2(f)–2(h); Supplementary Table 2).

### 3.2. Characteristics of Aortic Vascular Calcification in DN Rats

**Figure 3:**
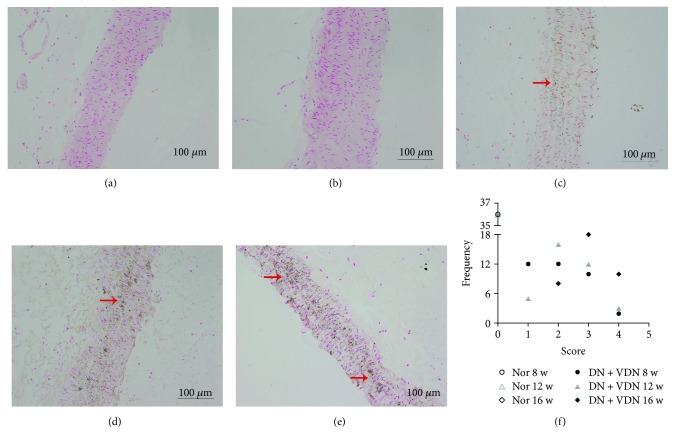
Histological analysis of aortic calcification with von Kossa staining. The von Kossa staining of aortic tissues from normal rats and DN + VDN rats. No deposition of calcium salts was observed in aortic tissues of normal rats at 8 (a) and 16 weeks (b). In contrast, rats in the DN + VDN group exhibited scattered, punctated deposition of calcium salts in the form of dark brown granules interspersed with the elastic fibers in arterial intima-media at the end of the 8th week (c); and a large quantity of aggregated deposits of calcium salts in vascular wall at the end of the 12th week (d); and finally formed a wide area of confluent calcification plaque at the end of the 16th week (e). (f) rats in the DN + VDN group had significantly higher vascular calcification scores than those of normal rats at 8, 12, and 16 weeks (*p* < 0.001). Data presented as mean ± standard deviation (SD). Nor group: normal controls; DN + VDN group: diabetic nephropathy rats with vitamin D3/nicotine-induced vascular calcification.

The absence of calcium salt deposition in aortic tissue of normal rats was revealed by von Kossa staining, irrespective of the time point (8 weeks, Figure 3(a); 16 weeks, Figure 3(b)). In contrast, rats in the DN + VDN group exhibited scattered, punctated deposition of calcium salts in the form of dark brown granules among the elastic fibers of the intima media at the end of the 8th week (Figure 3(c)); a large quantity of aggregated deposits of calcium salts was observed in vascular wall at the end of the 12th week (Figure 3(d)); finally, a wide area of confluent calcification plaque was observed at the end of the 16th week (Figure 3(e)). The distribution of scores in both groups is shown in Figure 3(f). The DN + VDN group had higher scores as compared to those of the Nor group (*p* < 0.05).

## 4. 3.3 mRNA Expressions of *α*-SMA, SM22*α*, Runx2, and ALP in Aortic Tissues

**Figure 4:**
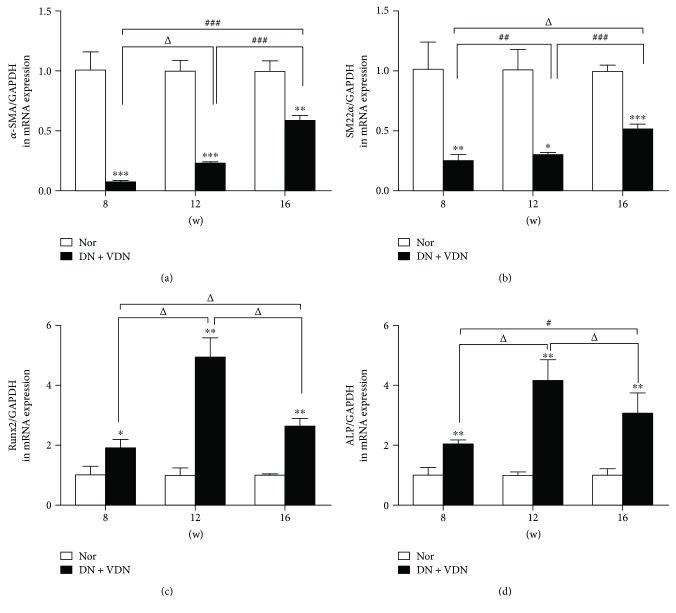
Quantitative assessment of mRNA expressions of *α*-SMA (a), SM22*α* (b), Runx2 (c), and ALP (d) in aortic tissues. Data presented as mean ± standard deviation (SD). ^∗^*p* < 0.05, ^∗∗^*p* < 0.01, and ^∗∗∗^*p* < 0.001 versus Nor group; ^Δ^*p* > 0.05, ^#^*p* < 0.05, ^##^*p* < 0.01, and ^###^*p* < 0.001. Nor group: normal controls; DN + VDN group: diabetic nephropathy rats with vitamin D3/nicotine-induced vascular calcification.

Real-time PCR showed that the mRNA expressions of *α*-SMA and SM22*α* in aortic tissue were significantly decreased at all time points (8, 12, and 16 weeks), as compared with the Nor group (*p* < 0.05, *p* < 0.01, or *p* < 0.001; Figures 4(a) and 4(b)). In contrast, the mRNA expressions of Runx2 and ALP in aortic tissues were upregulated after modeling (*p* < 0.05 and *p* < 0.01 versus Nor group; Figures 4(c) and 4(d)).

### 4.1. 3.4 Detection of *α*-SMA and Runx2 Levels by Immunohistochemical Analysis

**Figure 5:**
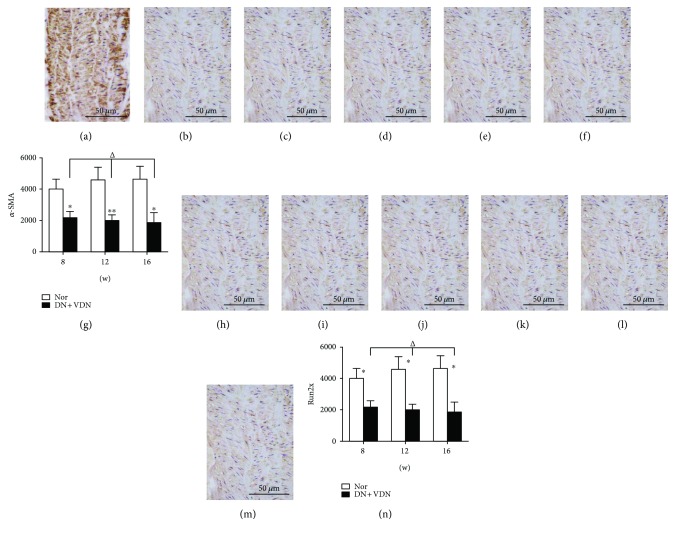
Detection of *α*-SMA and Runx2 levels by immunohistochemical analysis. *α*-SMA protein was extensively expressed in the aortic wall of normal rats at 8 weeks (a), 12 weeks (b), and 16 weeks (c), whereas the expression of *α*-SMA in the aortic tissue of DN + VDN group was significantly decreased at all time points, 8 weeks (d), 12 weeks (e), and 16 weeks (f). (g) Quantitative analysis of *α*-SMA was performed. Runx2 protein was weakly expressed in the wall of the aorta in normal rats at 8 weeks (h), 12 weeks (i), and 16 weeks (j). Its expression was significantly increased in the DN + VDN group at 8 weeks (k), 12 weeks (l), and 16 weeks (m). (n) Quantitative analysis of Runx2 was performed. Data presented as mean ± standard deviation (SD). ^∗^*p* < 0.05 and ^∗∗^*p* < 0.01 versus Nor group; ^Δ^*p* > 0.05. Nor group: normal controls; DN + VDN group: diabetic nephropathy rats with vitamin D3/nicotine-induced vascular calcification.

During the experiments, *α*-SMA protein was extensively expressed in the aortic wall of normal rats, and there was no significant difference at any of the time points (8, 12, and 16 weeks, *p* > 0.05; Figures 5(a)–5(c)). However, the expression of *α*-SMA in the aortic tissue of DN + VDN group was significantly lower than that in the Nor group at the same time point (*p* < 0.05 or *p* < 0.01 versus Nor group at 8, 12, and 16 weeks; Figures 5(d)–5(g)).

In addition, Runx2 protein was weakly expressed in the wall of the aorta in normal rats (8, 12, and 16 weeks; Figures 5(h)–5(j)), whereas its expression was significantly increased in the DN + VDN group at each time point (*p* < 0.05 versus Nor group at 8, 12, and 16 weeks; Figures 5(k)–5(n)).

## 5. 3.5 mRNA Expression of Notch1, RBP-Jk, Msx2, and Jagged1 in Aortic Tissues

**Figure 6:**
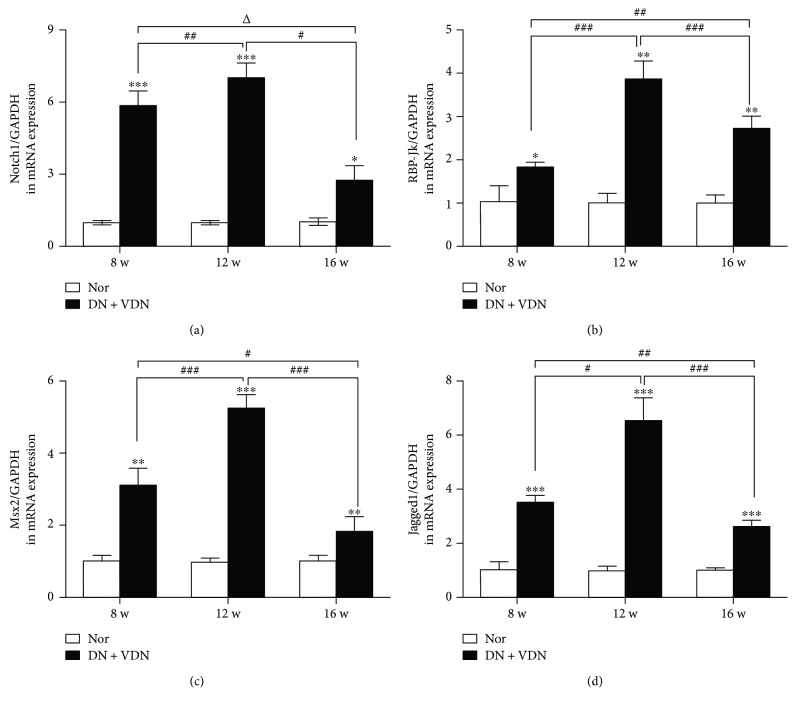
The mRNA expressions of Notch1 (a), RBP-Jk (b), Msx2 (c), and Jagged1 (d) in aortic tissues. Data presented as mean ± standard deviation (SD). ^∗^*p* < 0.05, ^∗∗^*p* < 0.01, and ^∗∗∗^*p* < 0.001 versus Nor group; ^Δ^*p* > 0.05, ^#^*p* < 0.05, ^##^*p* < 0.01, and ^###^*p* < 0.001. Nor group: normal controls; DN + VDN group: diabetic nephropathy rats with vitamin D3/nicotine-induced vascular calcification.

As compared with the normal rats, the mRNA expressions of Notch1, RBP-Jk, Msx2, and Jagged1 in aortic tissues were persistently increased till 12 weeks (*p* < 0.01 and *p* < 0.001* versus* Nor group) and then substantially decreased, although these were still higher than the normal levels (*p* < 0.05, *p* < 0.01, or *p* < 0.001; Figures 6(a)–6(d)).

### 5.1. 3.6 Detection of Notch1, RBP-Jk, Msx2, Jagged1, and N1-ICD Levels by Immunohistochemical Analysis

**Figure 7:**
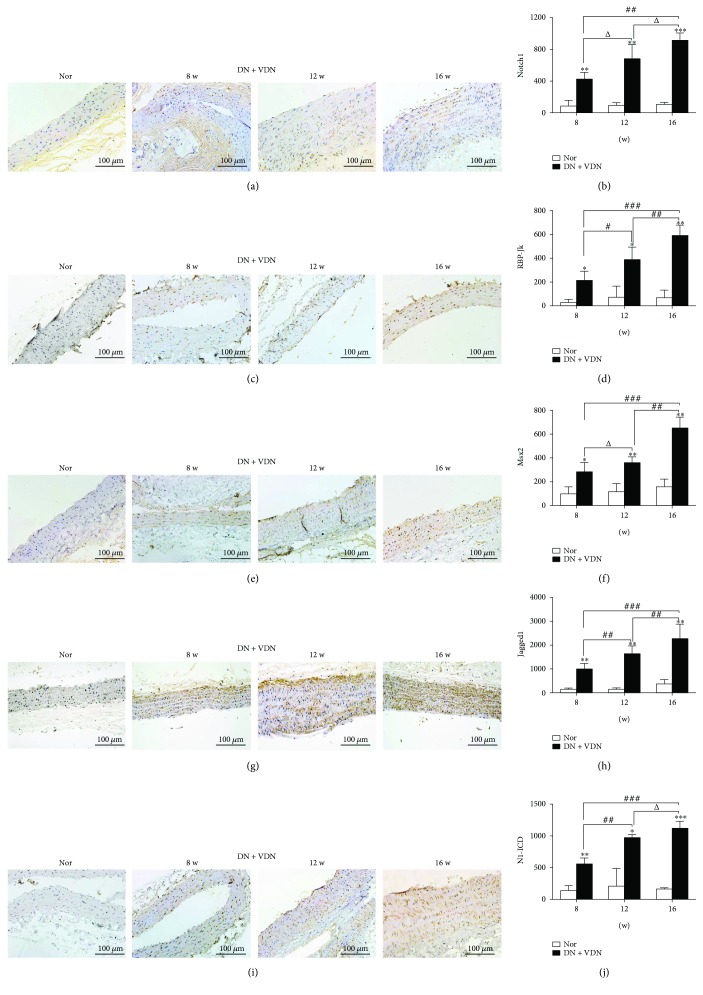
Detection of Notch1, RBP-Jk, Msx2, Jagged1, and N1-ICD levels by immunohistochemical analysis. The representative images and quantitative analysis of Notch1 (a-b), RBP-Jk (c-d), Msx2 (e-f), Jagged1 (g-h), and N1-ICD (i-j) in aortic tissues. The left panel of histological images: Nor group; right panel: DN + VDN group at 8, 12 , and 16 weeks, respectively. Data presented as mean ± standard deviation (SD). ^∗^*p* < 0.05, ^∗∗^*p* < 0.01, and ^∗∗∗^*p* < 0.001 versus Nor group; ^Δ^*p* > 0.05, ^#^*p* < 0.05, ^##^*p* < 0.01, and ^###^*p* < 0.001. Nor group: normal controls; DN + VDN group: diabetic nephropathy rats with vitamin D3/nicotine-induced vascular calcification.

Notch1, RBP-Jk, Msx2, Jagged1, and N1-ICD were barely expressed in the aortic wall of normal rats. However, the expressions of these proteins in the DN + VDN group were significantly increased at all time points as compared to that in the normal rats (8, 12, and 16 weeks; *p* < 0.05, *p* < 0.01, or *p* < 0.001; Figure 7). The positive staining was mainly distributed in the media of the aorta.

## 6. 4. Discussion

Intramuscular injection of vitamin D3 and/or intragastric administration of nicotine are commonly used to establish a rat model of vascular calcification [14, 16, 17]. High dose of vitamin D induces and exacerbates oxidative stress and results in extensive vascular calcification [18]. Nicotine releases catecholamines and promotes calcium deposition in blood vessels [16]. The combination of vitamin D3 and nicotine leads to a substantial increase (approximately more than 20-fold) in calcium content of the aorta in rats [16]. This model can simulate the pathological vascular calcification observed in diabetes, end-stage renal disease, and aging-related calcification [19], with high stability and excellent reproducibility.

In this study, we used a rat model of DN with concomitant vascular calcification by intraperitoneal injection of STZ followed by administration of vitamin D3 and nicotine. The rats exhibited typical characteristics of DN, including weight loss, persistent proteinuria, robust increase in serum BUN, and creatinine. Vitamin D can increase the serum calcium level, whereas nicotine toxicity induces calcium overload and even necrosis of VSMCs [19, 20]. The released calcium and other minerals are accumulated among the elastic fibers in the arterial walls. In this study, we observed a significant increase in serum calcium levels in model rats at all time points (8, 12, and 16 weeks); however, serum phosphorus level was not significantly different until 16 weeks. These results are consistent with the fact that hyperphosphatemia usually appears in the late stages of CKD [21]. The increase in serum calcium levels as well as calcium-phosphorus products can promote calcification. Hyperphosphatemia-related vascular calcification in patients with DN, end-stage renal disease, and metabolic syndrome occurs mainly in the arterial media [22, 23]. Histological studies of model rats demonstrated deposition of calcium salts, which indicates successful establishment of a rat model of vascular calcification. The effect of vascular calcification in patients with DN is not well understood. Thus, an in-depth understanding of the pathogenesis of vascular calcification in the context of DN will be of great significance and may facilitate the search for a new therapeutic target.

Vascular calcification resulting from deposition of calcium phosphorus or hydroxyapatite in the vessel wall is a complex pathological process regulated by a variety of regulatory factors and pathways. A number of studies have revealed that VSMCs play a central role in the initiation and progression of vascular calcification, during which they undergo phenotypic differentiation, for example, into osteoblast-like cells. The process is accompanied by overexpression of osteogenic markers such as Msx2, ALP, OPN, and type I collagen, which are involved in ectopic mineralization and bone formation [6, 8, 10]. In this study, real-time PCR showed a significant decrease in the mRNA expression of VSMC markers (*α*-SMA and SM22*α*) in aortic tissue of model rats; while the mRNA expressions of osteogenic markers (Runx2 and ALP) were remarkably increased, especially at 12 weeks. The expressions of these proteins were gradually restored to near normal levels over time although they were still lower (*α*-SMA and SM22*α*) or higher (Runx2 and ALP) than those in control animals, perhaps due to a compensatory mechanism of the body, which requires further investigation. These results were partially consistent with the immunohistochemical analysis, although some discrepancies between mRNA expressions of *α*-SMA and Runx2 and results of quantitative immunohistochemistry were observed. Further efforts are needed to explain these discrepancies. These results indicated that DN rats with vascular calcification exhibited mineral deposits in the arterial intima-media accompanied by phenotypic changes of VSMCs and activation of osteogenic factors.

Vascular calcification is associated with multiple signaling pathways, including BMP2/SMADs, receptor for advanced glycation end products (RAGE)/JAK2, Wnt/*β*-catenin, and Notch1 [10, 24]. Also, the involvement of Notch signaling pathway in DN has been elucidated [25, 26]. In this study, extensive arterial calcification in rats with DN was accompanied by upregulation of Notch1, RBP-Jk, Msx2, Jagged1, and N1-ICD proteins, which suggests the activation of Notch1 signaling pathway. There may be at least two possible mechanisms involved. Hyperglycemia can increase the expression of Notch1 protein and its downstream molecules involved in the pathogenesis of endothelial cell injury [27]. Culture of VSMCs *in vitro* with serum from diabetic patients was shown to induce an increase in calcium content of VSMCs [28]. In a study by Suga et al. [29], RAGE overexpression induced the expression of ALP and Msx2 and calcium deposition in human aortic VSMCs that were cultured with the sera of patients with diabetes; on the contrary, silencing of Msx2 effectively inhibited osteogenic differentiation of VSMCs, which indicates that activation of Notch/Msx2 signal pathway under high glucose induces osteogenic differentiation of VSMCs and contributes to aortic calcification. Secondly, in the late stage of DN, impaired renal function causes hyperphosphatemia which stimulates Notch1-RBP-Jk signaling pathway to induce the expression of target gene Msx2, resulting in osteogenic differentiation and mineralization of VSMCs [6, 11]. Interestingly, the expressions of target molecules in Notch1-RBP-Jk/Msx2 pathway might show a fluctuant increase, probably due to a synergistic or offsetting effect of various pathologic factors. For instance, hyperglycemia and hyperphosphatemia trigger the activation of Notch1-RBP-Jk/Msx2 pathway in DN to varying degrees and at different time periods, whereas the compensatory mechanism of the body may restore their levels. Therefore, further investigation is required to confirm and extend these results.

In conclusion, activation of Notch1-RBP-Jk/Msx2 signaling pathway may be involved in the development and progression of vascular calcification in DN. In subsequent research, efforts will be made to identify a novel therapeutic target, blocking of which leads to the prevention and treatment of vascular calcification in DN.

## Abbreviations

ALP: Alkaline phosphatase
*α*-SMA: Alpha-smooth muscle actin
BMP2: Bone morphogenetic protein 2
BUN: Blood urea nitrogen
CKD: Chronic kidney disease
N1-ICD: Intracellular Notch1 domain
OPN: Osteopontin
PBS: Phosphate-buffered saline
PCR: Polymerase chain reaction
RAGE: Receptor for advanced glycation end products
Runx2: Runt-related transcription factor2
Scr: Serum creatinine
SM22*α*: Smooth muscle 22 alpha
STZ: Streptozotocin
VDN: Vitamin D3 and nicotine
VSMC: Vascular smooth muscle cell.

## Supplementary Materials

Supplementary Materials Supplementary Table 1: The primers of target genes. Supplementary Table 2: Characteristics of rats at different time points.
