# Supplementary material for: The Involvement of Notch1-RBP-Jk/Msx2 Signaling Pathway in Aortic Calcification of Diabetic Nephropathy Rats
Source: J Diabetes Res. 2017 Dec 31;2017:8968523. doi: 10.1155/2017/8968523 (PMC5804331; doi:10.1155/2017/8968523)
Supplement: Supplementary Materials — Supplementary Table 1: The primers of target genes. Supplementary Table 2: Characteristics of rats at different time points. [file 8968523.f1.docx]

Supplementary Table 1: The primers of target genes.

| Genes |  | 5’-3’ | Size |
| --- | --- | --- | --- |
| Notch1 | Forward | TCTCACAGAATACAAGTGC | 132 bp |
|  | Reverse | CCACAGATGTATGAAGACTC |  |
| RBP-Jk | Forward | GAGCCATTCTCAGAGCCAAC | 232 bp |
|  | Reverse | TCCCCAAGAAACCACAAAAG |  |
| Msx2 | Forward | AAGGCAAAAAGACTGCAGGA | 163 bp |
|  | Reverse | GGATGGGAAGCACAGGTCTA |  |
| Jagged1 | Forward | GACTACGAGGGCAAGAACTG | 132 bp |
|  | Reverse | GTTGGAAGAGATATACCGCACC |  |
| α-SMA | Forward | AGAGGAGCATCCGACCTT | 130 bp |
|  | Reverse | AGAGGAGCATCCGACCTT |  |
| SM22α | Forward | GCGTGATTCTGAGCAAGTTG | 146 bp |
|  | Reverse | CGTGACTCCATAATCCTCAGC |  |
| Runx2 | Forward | CCCAACTTCCTGTGCTCC | 240 bp |
|  | Reverse | AGTGAAACTCTTGCCTCGTC |  |
| ALP | Forward | CAGAGAAAGAGAAAGACCCCAG | 147 bp |
|  | Reverse | CTGTCACTGTGGAGACGC |  |
| GAPDH | Forward | CCCCCAATGTATCCGTTGTG | 118 bp |
|  | Reverse | TAGCCCAGGATGCCCTTTAGT |  |

Supplementary Table 2: Characteristics of rats at different time points

|  | Time point  (weeks) | Nor | DN+VDN |
| --- | --- | --- | --- |
| Body weight, g | 8 | 361.81±5.28 | 315.31±3.79^***^ |
|  | 12 | 443.85±2.65 | 215.17±3.56^***^ |
|  | 16 | 483.22±22.31 | 179.76±3.64^***^ |
| Blood glucose, mmol**/**L | 8 | 8.11±2.19 | 27.52±5.74^**^ |
|  | 12 | 8.85±1.91 | 28.55±1.47^***^ |
|  | 16 | 8.06±0.57 | 27.43±1.95^***^ |
| 24-h urine protein excretion,  mg/24 h | 8 | 16.39±1.61 | 122.08±4.32^***^ |
|  | 12 | 22.86±1.98 | 152.00±15.93^***^ |
|  | 16 | 21.37±1.28 | 192.73±6.18^***^ |
| BUN, mmol/L | 8 | 6.02±0.18 | 8.46±0.21^***^ |
|  | 12 | 5.66±0.24 | 9.34±0.43^***^ |
|  | 16 | 5.65±0.25 | 9.67±0.11^***^ |
| Scr, μmol/L | 8 | 50.00±2.16 | 52.25±1.5 |
|  | 12 | 47.25±2.22 | 74.25±2.63^***^ |
|  | 16 | 36.00±2.58 | 78.75±2.50^***^ |
| Serum calcium | 8 | 2.35±0.20 | 2.73±0.12^*^ |
|  | 12 | 2.27±0.18 | 2.74±0.13^**^ |
|  | 16 | 2.53±0.06 | 2.83±0.11^**^ |
| Serum phosphorus | 8 | 2.52±0.32 | 2.91±0.16 |
|  | 12 | 2.48±0.37 | 2.91±0.22 |
|  | 16 | 2.69±0.42 | 3.63±0.30^*^ |
| Calcium-phosphorus product  (Ca×P) | 8 | 74.01±15.47 | 99.54±8.76^*^ |
|  | 12 | 70.14±16.03 | 98.53±2.82^*^ |
|  | 16 | 84.32±13.38 | 127.43±13.04^**^ |

* *p*<0.05, ** *p*<0.01, *** *p*<0.001 *vs.* Nor group.

Data presented as mean ± standard deviation.

BUN, blood urea nitrogen; Scr, serum creatinine.
